# Effect of protease supplementation on amino acid digestibility of soybean meal fed to growing-finishing pigs in two different ages

**DOI:** 10.1093/jas/skae345

**Published:** 2024-11-07

**Authors:** Gabriela M Galli, Crystal L Levesque, Vinicius S Cantarelli, Rhuan F Chaves, Claudia C Silva, Vitor B Fascina, Jorge Y Perez-Palencia

**Affiliations:** Department of Animal Science, South Dakota State University, Brookings, SD 57007, USA; Department of Animal Science, Federal do Rio Grande do Sul, Porto Alegre, RS, Brazil; Department of Animal Science, South Dakota State University, Brookings, SD 57007, USA; Animalnutri, Patos de Minas, Brazil; Animalnutri, Patos de Minas, Brazil; dsm-firmenich, São Paulo, Brazil; dsm-firmenich, São Paulo, Brazil; Department of Animal Science, South Dakota State University, Brookings, SD 57007, USA

**Keywords:** exogenous enzymes, feed formulation, pig age, protein, soybean meal, swine

## Abstract

This study was conducted to investigate the effect of protease inclusion level in two different ages on the apparent (**AID**) and standardized (**SID**) ileal digestibility of crude protein **(CP)** and amino acids **(AAs)** in soybean meal **(SBM)** fed to growing-finishing pigs. Ten cannulated pigs (21 ± 2 kg) were assigned to experimental diets in a duplicate 5 × 5 Latin square design. In phase I (23 to 30 kg-pigs, 90 ± 17 d of age), ileal digesta was collected in five periods of 7 d (5 d adaptation and 2 d ileal digesta collection). In phase II, (50 to 65 kg-pigs, 140 ± 17 d of age), ileal digesta was collected in 5 more periods of 7 d. For both phases, a corn starch–based diet was formulated with SBM as the sole source of CP and AA and containing titanium as an indigestible marker. Protease was supplemented at 0, 15,000, 30,000, and 45,000 NFP/kg of feed (0, 25, 50, and 75 g/ton of ProAct 360). A nitrogen-free diet was used to estimate basal ileal endogenous AA losses. Pigs were fed at 4% of their body weight, which was adjusted at the end of each period. Orthogonal polynomial contrasts were used to determine the linear and quadratic effects of dietary protease supplementation in each phase. In phase I, increasing levels of protease resulted in a linear increase (*P* < 0.10) in SID for the 7/11 indispensable AA (Except Arg, His, Met + Cys, and Trp) and the average of all dispensable AA. In phase II, the SID of Ile, Leu, Met, Met + Cys, Val, the average of all indispensable AA, and 4/7 dispensable AA were quadratically increased (*P* < 0.10). In most cases, supplementation with 30,000 NFP/kg of feed (50 g/ton) resulted in the greatest increase in AA digestibility. However, the linear response in phase I for some AA suggests that diets for younger pigs could be supplemented with a greater level (45,000 NFP/kg or 75 g/ton of feed). Interestingly, younger pigs had consistently increased (*P* < 0.10) SID of CP and 15/18 AA (Except Arg, Cys, and Ser), being ~5.6% greater for indispensable AA when compared to older pigs. In conclusion, dietary protease supplementation can increase the SID of AA in SBM in both growing and finishing periods. Pig age can potentially influence AA digestibility, possibly related to a greater ileal endogenous AA flow in younger pigs. However, this fact warrants further investigation.

## Teaser

Supplementation with protease (50 g/ton or 30,000 NFP/kg of feed) can be used in growing-finishing pig feed programs to increase amino acid digestibility in soybean-meal-containing diets. Pig age could influence AA digestibility in which younger pigs had greater SID of AA compared to older pigs.

## Introduction

Feed costs constitute 65% to 75% of the total cost of pig production. Furthermore, public pressure to reduce the environmental impact of livestock has increased, and there is a need to include agricultural additives in animal diets to support greater efficiency in the use of current feed ingredients (Oxenboll et al., 2011; Wang et al., 2020). The main protein source in pig diets is soybean meal **(SBM)**, which is among the most expensive ingredient in swine diets (Min et al., 2019); although SBM contains a relatively high level of anti-nutritional factors (ANFs) and non-starch polysaccharides, which negatively impact nutrient digestion and contribute to variations in the nutritional value of SBM (Liener, 1994; Wang et al., 2011). These ANFs in soybeans include trypsin inhibitor, antigens (glycinin and conglycinin), lectins, phytoestrogens, oligosaccharides, and phytins (Liener, 1994; Jo et al., 2012). These ANFs increase pancreatic weight and compromise energy, protein, and amino acid (**AA**) digestibility (Wedekind et al., 2020). Heat treatment can reduce some of these factors, but not others, such as Bowman-Birk and Kunitz inhibitors of chymotrypsin and trypsin (Friedman and Brandon, 2001). However, the thermal process can reduce the availability of some AA, such as lysine, arginine (McDonald et al., 2011), and peptides (Sklan et al., 1975).

Therefore, the swine industry is searching for additives that contribute to the reduction in feed costs by improving nutrient digestion and absorption and maximizing growth performance. The inclusion of exogenous proteases as a nutritional strategy has led to improved nutrient utilization, reduced feed costs, and reduced manure nitrogen and inorganic phosphorus content (Kim et al., 2020a). Protease supplementation improves nutrient digestibility and reduces NH_3_ gas emissions (Nguyen et al., 2018). Proteases also improves growth (Min et al., 2019) and intestinal health (Perez-Palencia et al., 2021). Most studies in the literature utilized enzyme blends containing proteases and other enzymes, such as carbohydrates. Few studies have reported the supplementation of corn–SBM-based diets with proteases alone (Lee et al., 2018; Jang et al., 2021) or tested different levels of supplementation. In addition, the effectiveness of protease in swine diets can be variable and has been associated with the type of protease used, dose, feed ingredients used in formulation, and potentially animal age/category (Cowieson and Roos, 2016; Lee et al., 2018).

Thus, a greater understanding of how protease supplementation impacts nutrient availability as influenced by protein source, level, and pig age will provide critical information necessary for the optimal utilization of proteases in swine feeding programs that enhance growth performance and increase producers’ profitability. Therefore, this study aimed to evaluate the effect of protease inclusion level in 2 different ages on the apparent (**AID**) and standardized (**SID**) ileal digestibility of crude protein (**CP**) and AA in SBM fed to growing-finishing pigs.

## Materials and Methods

The study protocol was approved by the South Dakota State University Institutional Animal Care and Use Committee (IACUC Protocol No. 2104-020A).

### Animals and experimental design

Ten barrows with an initial body weight (**BW**) of 23 ± 2 kg, offspring of PIC Cambrough sows and Duroc boars were selected and surgically fitted with a simple T-cannula in the distal ileum. Pigs were adapted to individual housing equipped with a partially slatted floor, dry feeder, and water/nipple cup in a temperature-controlled room. The facility was operated on mechanical ventilation, with temperatures set at 21, 19, and 18 °C during the first 5 wk and 18, 15, and 14 °C in the last 5 wk of the study.

The experiment was conducted according to a duplicate 5 × 5 Latin square design, such that each pig received each of the five experimental diets. Feeding levels were based on pig BW (4% of BW) and adjusted for individual pigs at the beginning of each collection period. The diets were administered in mash form twice a day (8:00 a.m. and 4:00 p.m.), and water was provided ad libitum throughout the experimental period. For each 7-d period, the initial 5 d were considered a diet adaptation period followed by 12 h of continuous ileal digesta collections on days 6 and 7 (8 a.m. to 8 p.m.) using a plastic bag fixed to the cannulas (Kim et al., 2020b). Each plastic bag contained 5 mL of 10% formic acid to limit microbial growth (Htoo et al., 2007; Ricke et al., 2020), which was replaced when it reached 50% to 70% of its capacity. Digesta samples from each pig were mixed and sub-sampled at the end of each collection period.

Ileal digesta is collected over 2 experimental phases of 5 wk each. In phase I, the pig BW ranged from 23 to 30 kg, with an average age of 90 d. In phase II, the pig BW ranged from 50 to 65 kg, with an average age of 140 d. Pigs were allowed a 2-wk interval between phases to create a suitable difference in BW and age. In this interval, the pigs were fed ad libitum (Supplementary Table S1).

### Experimental diets

A corn–starch-based diet was formulated with SBM as the sole source of AA and supplemented with 25, 50, and 75 g/ton of protease to create 3 additional dietary treatments consisting of protease supplementation at 15,000, 30,000, and 45,000 NFP/kg of feed, respectively (ProAct 360, dsm-firmenich, São Paulo, Brazil). This protease was a novel subtilisin protease from *Bacillus* sp. produced in *Bacillus licheniformis* (Cupi et al., 2022). This is a sfericase protease, an endopeptidase from the serine protease family, subtilisin subfamily A, MEROPS ID S08.113 (Rawlings et al., 2014). Activity for this protease is defined in New Feed Protease (NFP) units, which measures the enzyme amount required to hydrolyze 1 µmol of para-nitroaniline (pNA) from 1 M substrate Suc-Ala-Ala-Pro-PhepNA per minute at pH 9.0 and 37 °C (Cupi et al., 2022).

An N-free diet was used to estimate basal ileal endogenous AA losses required to calculate SID. Minerals and vitamins were added to the diets according to NRC (2012) nutrient requirement estimates for growing pigs (Table 1). Titanium oxide (0.3% of the diet) was included in the diets as an indigestible marker to determine nutrient digestibility. Diets were mixed by period using the same ingredients, and the same batch of SBM was used to mix all the experimental diets in this study. During the interval period, all pigs were fed a standard diet that met or exceeded the growing pig requirement estimates (NRC, 2012). The SBM for this study was obtained from South Dakota Soybean processors (100 Caspian Ave, Volga, SD 57071, USA) and represented SBM from the US Midwest area.

**Table 1.** Experimental diets formulation and calculated composition (as-fed basis)

Ingredient	N-free diet	Control^1^
Cornstarch	66.8	55.6
Soybean meal	0.00	30.0
Sucrose/sugar	20.0	10.0
Cellulose (sulkafloc)	5.00	0.00
Soybean oil	4.00	0.80
Monocalcium phosphate	1.72	1.18
Limestone	1.15	1.12
Salt	0.70	0.65
Titanium oxide	0.30	0.30
Swine mineral premix^2^	0.15	0.15
Swine larvicide^3^	0.13	0.13
Swine vitamin premix^4^	0.05	0.05
*Calculated composition*		
ME, kcal/kg	3430	3491
Crude protein, %	0.00	14
SID lysine, %	0.00	0.82
SID Met, %	0.00	0.18
SID, Met + Cys, %	0.00	0.37
SID THR, %	0.00	0.47
SID TRP, %	0.00	0.18
SID ILEU, %	0.00	0.60
SID LEU, %	0.00	0.96
SID VAL, %	0.00	0.59
SID HIS, %	0.00	0.33
SID PHE, %	0.00	0.64
Calcium, %	0.70	0.70
Phosphorus, %	0.37	0.47
ATTD P, %	0.31	0.29
STTD P, %	0.33	0.33
Sodium, %	0.28	0.28
Cloride, %	0.41	0.53

^1^Protease was included on top at 25, 50, and 75 g/ton in the control diet to create 3 additional dietary treatments consisting of protease supplementation at 15,000, 30,000, and 45,000 NFP/kg of feed, respectively (ProAct 360, dsm-firmenich, São Paulo, Brazil).

^2^J & R Distributing Inc.,USA. Minimun provided per kilogram of diet: copper 16.5 mg, manganese 44.1 mg, selenium 0.3 mg, zinc 165 mg.

^3^Rabon 7.76 oral larvicide premix active ingredient tetrachlorvinphos manufactured for Elanco US.

^4^J & R Distributing Inc., USA. Minimun provided per kilogram of diet: Vitamin A 11,023 IU, vitamin D3 1,653 IU, vitamin E 55 IU; vitamin B12 0.04 mg, menadione 4.41 mg, riboflavin 9.92 mg, d-pantothenic acid 60.63 mg, thiamine 3.31 mg, niacin 55.11 mg, vitamin B6 3.31 mg, folic acid 1.10 mg, biotin 0.17 mg.

### Chemical analysis

A sample of SBM used in this study was analyzed for proximate analysis, trypsin inhibition, and a complete AA profile, as well as a sample of corn starch (Table 2).

**Table 2.** Analyzed composition of ingredients and experimental diets (as-fed basis)

Nutrient	Corn starch	Soybean meal^1^	N-free diet	Control	Protease 25 g/Ton	Protease 50 g/Ton	Protease 75 g/Ton
*Proximate analysis*							
Protease activity, NFP/kg^2^	–	–	0.00	0.00	12,290	41,690	57,325
DM, %	89.2	90.2	92.3	91.2	91.0	91.2	91.0
CP, %	0.60	44.3	0.45	13.2	15.1	14.4	13.1
GE, Kcal/kg	–	–	3,852	3,831	3,804	3,819	3,798
Crude Fat, %	0.00	1.32	3.75	1.10	0.65	0.93	1.16
Crude Fiber, %	0.02	3.24	2.01	1.19	1.11	1.06	1.06
Ash, %	0.10	5.95	2.89	5.37	4.73	5.79	4.49
NDF^3^, %	0.07	9.31	0.78	2.54	2.47	2.44	2.92
ADF^4^, %	0.00	4.84	0.02	1.65	1.89	1.54	1.60
Calcium, ppm	–	–	6,850	7,030	6,930	6,900	7,080
Phosphorus, ppm	–	–	2,910	3,130	3,140	3,150	3,040
Titanium, %	–	–	0.18	0.18	0.19	0.19	0.18
*Indispensable AA %*							
Arg	0.01	3.05	0.01	0.86	0.88	0.9	0.92
His	0.01	1.20	0.00	0.33	0.34	0.38	0.35
Ile	0.01	2.12	0.01	0.60	0.61	0.69	0.63
Leu	0.03	3.39	0.03	0.97	1.00	1.13	1.03
Lys	0.01	2.87	0.01	0.82	0.84	0.85	0.86
Met	0.01	0.63	0.00	0.16	0.17	0.20	0.18
Phe	0.02	2.23	0.01	0.64	0.66	0.74	0.68
Thr	0.01	1.74	0.01	0.49	0.5	0.57	0.52
Trp	0.01	0.66	0.01	0.20	0.21	0.20	0.20
Val	0.02	2.20	0.03	0.63	0.64	0.71	0.65
Total	0.14	20.0	0.12	5.70	5.85	6.37	6.02
*Dispensable AA %*							
Ala	0.02	1.93	0.02	0.56	0.57	0.64	0.58
Asp	0.03	4.99	0.02	1.42	1.47	1.66	1.51
Cys	0.00	0.68	0.00	0.18	0.19	0.22	0.18
Glu	0.04	8.01	0.03	2.31	2.39	2.70	2.45
Gly	0.01	1.87	0.01	0.53	0.55	0.62	0.56
Ser	0.01	1.89	0.01	0.55	0.60	0.67	0.61
Tyr	0.01	1.28	0.01	0.39	0.41	0.47	0.42
Tau	0.03	0.09	0.22	0.24	0.23	0.23	0.23
Pro	0.03	2.49	0.06	0.71	0.66	0.75	0.69
Total	0.18	23.3	0.38	6.92	7.10	8.00	7.27
Total AA mean	0.32	43.4	0.50	12.62	12.9	14.4	13.3

^1^Trypsin inhibitor activity in the soybean meal sample was 4.77 ± 0.67 TUI. Activity is expressed as trypsin units inhibited (TUI) per milligram of sample.

^2^Analyzed as described by Vieira et al. (2023).

^3^Neutral detergent fiber.

^4^Acid detergent fiber.

Ileal digesta and experimental diet samples were analyzed for CP, AA, and titanium. The ileal digesta samples were sub-sampled and then freeze-dried. Ileal digesta and diet samples were ground to pass through a 0.5-mm screen using a mill grinder (Retsch zm 200, ring sieve size: 0.75 mm) before chemical analysis.

To measure CP and AA concentrations, samples were analyzed at a commercial laboratory (University of Missouri, Columbia MO) using method 990.03; AOAC International, 2007 and method 982.30 E; AOAC, 2005. The titanium concentration was analyzed following the procedure of Myers et al. (2004), and the absorbance was measured using a SpectraMAX 190 plate reader at a wavelength of 408 nm.

The analyzed mean value for titanium in diets and ileal samples were used to calculate the AID following equation (Stein et al., 2007; Kong and Adeola, 2014):


$$\begin{array}{l} AID,\%=100-\left[100\times \left(\frac{Ti_{d}\times AA_{i}}{Ti_{i}\times AA_{d}}\right)\right] \end{array}$$

where Ti_d_ is the concentration of titanium in the diet, Ti_i_ is the  concentration of titanium in ileal sample, AA_i_ is the concentration of the AA in ileal sample, and AA_d_ is theconcentration of the AA in the diet.

The basal endogenous losses of CP and AA were determined as the average from pigs fed the N-free diet and used to calculate SID of CP and AA by correcting AID values for basal endogenous losses of CP and AA according to the following equation (Stein et al., 2007; Kong and Adeola, 2014):


$$\begin{array}{l} SID,\%=AID+\left[100\times \left(\frac{Basal\;endogenous\;AA\;loss}{AA\;intake}\right)\right] \end{array}$$

### Statistical analysis

The UNIVARIATE procedure in SAS (SAS Inst. Inc., Cary, NC) was used to confirm the homogeneity of the variance and analyze the outliers. Orthogonal polynomial contrasts were used to determine the linear and quadratic effects of protease supplementation in each phase. The model included the fixed effects of dietary treatment and random effects of pigs and period. Contrast statements were used to compare AA digestibility among specific treatments (i.e., 0% inclusion vs. protease supplementation). The AID, SID, and basal ileal endogenous losses data were analyzed using PROC TTEST procedure of SAS considering the two ages as main effect. For all statistical tests, differences were considered at *P* ≤ 0.05 and tendency 0.05 *P *≤ 0.10.

## Results

### Phase I (23 to 30 kg; 90 d old)

Increasing level of protease supplementation resulted in a linear increase (*P *< 0.05) in AID of Ile, Leu, Met, Phe, Thr, and Asp with a tendency for Lys, Tyr, and the average of all dispensable AA (Table 3). Regardless of the supplementation level, protease increased (*P* < 0.10) the AID of Ile, Leu, Lys, Met, Met + Cys, Phe, Thr, Val, Asp, and the average of all indispensable AA compared with the control (Table 3).

**Table 3.** Effects of dietary protease supplementation on apparent ileal digestibility (%) of the protein and amino acids of soybean meal fed to growing-finishing pigs (23 to 30 kg/90 d old), phase I

Item	Protease inclusion, g/ton				SEM	*P*-value		
	0	25	50	75		Linear	Quadratic	CON vs. protease^1^
CP	79.6	80.3	86.7	80.2	3.18	0.71	0.48	0.63
*Indispensable AA*								
Arg	80.6	81.6	81.7	83.0	2.35	0.54	0.95	0.62
His	82.4	83.9	85.4	85.8	1.42	0.13	0.75	0.19
Ile	80.6	81.6	81.7	83.0	1.31	0.05	0.31	0.05
Leu	78.2	80.3	83.1	82.2	1.35	0.04	0.32	0.04
Lys	78.5	81.7	82.6	83.1	1.65	0.07	0.42	0.08
Met	82.7	84.2	88.5	87.7	1.50	0.02	0.48	0.04
Met + Cys	68.9	71.1	77.0	72.6	2.10	0.12	0.17	0.09
Phe	80.0	82.0	84.5	83.7	1.31	0.05	0.34	0.05
Thr	72.3	77.7	84.1	81.2	2.39	0.03	0.22	0.03
Trp	83.9	84.9	83.3	84.3	1.29	0.96	0.99	0.87
Val	73.2	75.9	77.9	77.0	1.57	0.10	0.30	0.07
Mean	78.1	80.3	82.2	81.9	1.43	0.22	0.71	0.09
*Dispensable AA*								
Ala	55.0	56.9	57.7	60.3	3.40	0.28	0.91	0.41
Asp	76.6	79.1	81.8	81.3	1.72	0.03	0.36	0.04
Cys	55.0	58.0	65.5	57.4	3.27	0.31	0.10	0.16
Glu	86.0	87.6	89.7	88.3	1.72	0.22	0.37	0.18
Gly	4.10	3.50	7.40	11.2	8.11	0.40	0.61	0.81
Ser	71.4	70.7	75.8	74.4	2.09	0.12	0.87	0.33
Tyr	79.1	80.4	85.7	82.8	1.94	0.08	0.29	0.10
Mean	64.9	65.9	69.5	68.6	2.58	0.08	0.46	0.31
Total AA mean	60.2	61.4	63.4	64.8	3.11	0.22	0.97	0.37

^1^Contrast: control diet (without protease supplementation) vs. protease supplementation (25 to 75 g/ton). Each dietary treatment had 10 observations.

The SID of Ile, Leu, Lys, Met, Phe, Thr, Val, Asp, and the mean indispensable AA linearly increased as a result of protease supplementation (*P* < 0.10). Supplementation with protease increased (*P* < 0.10) the SID of Ile, Leu, Lys, Met, Phe, Thr, Val, and Asp compared to the control diet (Table 4).

**Table 4.** Effects of dietary protease supplementation on standardized ileal digestibility (%) of the protein and amino acids of soybean meal fed to growing-finishing pigs (23 to 30 kg/90 d old), phase I

Item	Protease inclusion, g/ton				SEM	*P*-value		
	0	25	50	75		Linear	Quadratic	CON vs. protease^1^
CP	82.6	83.1	88.5	84.0	3.68	0.63	0.58	0.61
*Indispensable AA*								
Arg	88.5	88.4	88.2	90.1	3.23	0.73	0.75	0.90
His	86.4	87.8	88.8	89.8	1.61	0.16	0.92	0.24
Ile	84.2	86.4	88.1	88.0	1.41	0.06	0.43	0.06
Leu	81.9	83.9	86.2	85.8	1.46	0.04	0.40	0.05
Lys	82.0	86.5	86.7	88.2	1.96	0.03	0.42	0.02
Met	87.0	88.6	92.0	91.9	1.82	0.03	0.63	0.07
Met + Cys	74.7	77.1	82.1	78.6	2.37	0.13	0.23	0.11
Phe	83.9	85.8	88	87.5	1.43	0.05	0.41	0.06
Thr	75.4	80.1	85.3	83.3	2.64	0.03	0.25	0.03
Trp	90.5	91.6	90.3	91.1	1.61	0.93	0.94	0.78
Val	79.0	81.4	83.1	82.7	1.70	0.09	0.40	0.08
Mean	83.4	85.5	87.1	87.3	1.59	0.08	0.56	0.10
*Dispensable AA*								
Ala	64.1	65.4	65.7	69.2	3.30	0.30	0.74	0.50
Asp	79.9	82.5	84.7	84.8	1.64	0.03	0.44	0.03
Cys	62.5	62.5	72.2	65.4	3.16	0.29	0.13	0.16
Glu	88.5	90.0	91.7	90.7	1.81	0.27	0.47	0.24
Gly	22.9	17.9	25.3	30.0	7.94	0.40	0.52	0.86
Ser	79.0	78.2	82.6	81.7	2.04	0.16	0.99	0.41
Tyr	84.2	85.6	90.6	87.9	1.99	0.11	0.34	0.12
Mean	73.1	73.9	77.1	76.7	2.43	0.22	0.81	0.34
Total AA mean	69.8	70.2	72.1	74.3	3.15	0.24	0.76	0.47

^1^Contrast: control diet (without protease supplementation) vs. protease supplementation (25 to 75 g/ton). Each dietary treatment had 10 observations.

### Phase II (50 to 65 kg; 140 d old)

Increasing levels of protease supplementation resulted in a quadratic increase (*P *< 0.05) in the AID of Leu, Met, Met + Cys, Val, Cys, Glu, Tyr, and the average of all indispensable and total AA. The highest values of AID of Leu, Met, Met + Cys, Val, Cys, Glu, Tyr, and the average of all indispensable and total AA were observed at 50 g/ton of protease while 75 g/ton resulted in the lowest values. In addition, there was a tendency for quadratic increase (*P *< 0.10) in the AID for Arg, Ile, Gly, and the average of all dispensable AA. The highest values of AID for Arg, Ile, Gly, and the average of all dispensable AA were observed with the supplementation of 50 g/ton of protease, while the lowest values were observed with 75 g/ton (Table 5).

**Table 5.** Effects of dietary protease supplementation on apparent ileal digestibility (%) of the protein and amino acids of soybean meal fed to growing-finishing pigs (50 to 65 kg/140 d old), phase II

Item	Protease inclusion, g/ton				SEM	*P*-value		
	0	25	50	75		Linear	Quadratic	CON vs. protease^1^
CP	76.1	78.7	80.2	75.3	2.48	0.96	0.39	0.67
*Indispensable AA*								
Arg	78.1	82.7	81.7	78.4	1.84	0.99	0.08	0.19
His	82.1	84.2	84.1	80.9	1.53	0.64	0.15	0.61
Ile	80.2	81.8	83.6	78.7	1.40	0.70	0.06	0.53
Leu	79.0	80.9	83.9	78.5	1.39	0.86	0.03	0.24
Lys	69.8	72.8	73.6	70.3	1.65	0.78	0.12	0.21
Met	82.4	84.7	86.2	81.0	1.56	0.75	0.05	0.44
Met + Cys	71.7	75.0	77.5	70.1	1.88	0.81	0.02	0.32
Phe	80.3	82.2	81.6	79.3	1.40	0.63	0.21	0.67
Thr	75.1	78.0	78.4	75.4	1.94	0.90	0.29	0.47
Trp	83.2	83.6	88.1	85.1	1.51	0.18	0.33	0.23
Val	73.9	75.9	78.1	71.0	1.63	0.47	0.03	0.60
Mean	77.6	79.9	81.1	77.1	1.44	0.80	0.04	0.35
*Dispensable AA*								
Ala	51.3	56.4	55.2	50.7	2.72	0.84	0.15	0.35
Asp	77.6	79.6	79.7	76.4	1.62	0.69	0.17	0.65
Cys	60.9	65.4	68.7	59.1	2.32	0.87	0.02	0.27
Glu	83.4	87.2	86.9	81.3	1.87	0.50	0.04	0.48
Gly	1.80	9.90	8.40	6.70	5.71	0.60	0.06	0.43
Ser	76.2	78.0	79.9	76.4	1.49	0.74	0.14	0.35
Tyr	75.8	81.2	81.5	76.8	1.43	0.67	0.01	0.04
Mean	64.1	68.7	69.3	62.9	2.15	0.96	0.08	0.31
Total AA mean	58.0	62.8	62.8	56.4	2.29	0.70	0.05	0.36

^1^Contrast: control diet (without protease supplementation) vs. protease supplementation (25 to 75 g/ton). Each dietary treatment had 10 observations.

The SID of Ile, Leu, Met, Met + Cys, Val, and the average of all indispensable AA quadratically increased (*P* < 0.10) as a result of protease supplementation (Table 6). In addition, the SID of the majority of dispensable AA quadratically increased (*P* < 0.10). Supplementation with protease increased (*P* < 0.05) the AID and SID of Tyr when compared to that of the control.

**Table 6.** Effects of dietary protease supplementation on standardized ileal digestibility (%) of the protein and amino acids of soybean meal fed to growing-finishing pigs (50 to 65 kg/140 d old), phase II

Item	Protease inclusion, g/ton				SEM	*P*-value		
	0	25	50	75		Linear	Quadratic	CON vs. protease^1^
CP	78.2	80.9	82.5	77.4	2.6	0.84	0.37	0.72
*Indispensable AA*								
Arg	84.9	89.5	87.9	84.9	2.25	0.89	0.13	0.28
His	83.7	85.8	85.2	82.2	1.63	0.52	0.16	0.70
Ile	80.7	82.2	83.3	79.0	1.45	0.58	0.08	0.64
Leu	79.4	81.2	83.6	78.7	1.43	0.97	0.04	0.29
Lys	76.0	78.3	79.1	75.8	1.94	0.98	0.20	0.40
Met	82.7	85.0	85.9	81.2	1.68	0.64	0.07	0.54
Met + Cys	73.7	77.0	78.7	71.9	1.95	0.71	0.02	0.36
Phe	80.6	82.4	81.5	79.5	1.45	0.55	0.24	0.75
Thr	75.6	78.2	78.2	75.9	1.98	0.95	0.31	0.50
Trp	83.5	84.0	87.8	84.8	1.65	0.31	0.29	0.30
Val	75.6	77.5	79.0	72.6	1.67	0.36	0.03	0.69
Mean	80.2	82.3	83.1	79.4	1.49	0.69	0.06	0.43
*Dispensable AA*								
Ala	57.3	62.1	60.5	56.4	2.93	0.77	0.18	0.41
Asp	77.8	79.7	79.5	76.4	1.69	0.61	0.19	0.73
Cys	64.6	69.0	71.5	62.7	2.39	0.78	0.02	0.30
Glu	82.5	86.3	85.7	80.2	2.02	0.45	0.04	0.49
Gly	14.0	25.5	23.3	8.8	6.10	0.55	0.06	0.46
Ser	80.2	82.0	83.5	80.3	1.54	0.82	0.15	0.39
Tyr	78.3	83.3	83.2	78.9	1.51	0.82	0.01	0.06
Mean	68.0	72.9	72.0	66.7	2.42	0.82	0.08	0.34
Total AA mean	65.7	70.4	69.9	63.8	2.46	0.61	0.05	0.40

^1^Contrast: control diet (without protease supplementation) vs. protease supplementation (25 to 75 g/ton). Each dietary treatment had 10 observations.

### Age effects on AA digestibility

There was increased (*P *< 0.10) AID of Lys, Met, Ala, Cys, Glu, Ser, and Tyr in younger pigs (90 d of age) compared with older pigs (140 d of age; Supplementary Table S2). The SID of CP and most AA (except Arg, Cys, and Ser) was also increased in younger pigs than in older pigs (Supplementary Table S3). The increase in the SID of CP in younger pigs compared to older pigs was 5.9% on average, and 11.0%, 7.3%, 5.2%, 6.8% for the first four limiting AA in swine (Lys, Met, Thr, and Trp, respectively). Ileal endogenous losses of AA were greater (*P* < 0.09) in younger pigs than in older pigs for His, The, Try, Val, Lanthionine, Ser, and Tau (Supplementary Table S4).

## Discussion

This study was conducted to evaluate the effects of protease inclusion level in 2 different ages on the AID and SID of CP and AA in SBM fed to growing-finishing pigs. The hypothesis tested in this study states that increasing levels of protease results in greater AA digestion regardless of pig age/phase. Overall, the SID of the majority of indispensable AA and the average of all dispensable AA was increased linearly in phase I while in phase II the SID of Ile, Leu, Met + Cys, Val, Cys, and the average of all dispensable AA was quadratically increased. The effectiveness of proteases in pigs can vary because of differences in ingredients, age of pigs, dose, enzyme products, or interactions with other enzymes (Cowieson and Roos, 2016; Lee et al., 2018). In most cases, supplementation with 30,000 NFP/kg of feed (50 g/ton) resulted in the greatest improvement in AA digestibility, increasing the SID of CP by 5.5% and the first 3 limiting AA in swine: Lys (4.9%), Met (4.8%), and Thr (8.2%).

In this study, protease inclusion (50 g/ton or 30,000 NFP/kg of feed) in a corn starch-SBM-based diet improved the total indispensable and dispensable AA digestibility by 4.0% and 5.7%, respectively. Similar improvements in CP and AA digestibility were reported by Guggenbuhl et al. (2012), Song et al. (2022), and Zhang et al. (2023), using the same protease as our study (ProAct), an acid-stable protease, but supplementing 15,000 NFP/kg, 15,000 NFP/kg, and 11,250 NFP/kg, respectively, in a complete corn–SBM-based diet fed to weaned pigs. Pan et al. (2016) using corn–SBM-based diet in growing pigs reported slightly greater CP and AA digestibility improvements when supplementing 200 g/ton of coated compound proteases contained acidic, neutral, and alkaline proteases with total protease activity of 8,000 units/g. It is speculated that the improved digestibility in this study was due to protease work in different intestinal segments (small intestine) to cover the deficit of endogenous enzymes.

Collectively, exogenous protease supplementation can contribute to greater CP and AA digestibility values by improving the hydrolysis of proteins into smaller peptides, which facilitates their absorption and utilization, particularly when including SBM in the diets, as SBM proteins are less degraded by pancreatic proteases (Guggenbuhl et al., 2012; Leet et al., 2018). In addition, exogenous protease supplementation can stimulate nutrient digestion by minimizing the negative effects of ANFs, especially when SBM is included in the diets (Jo et al., 2012). In poultry, similar reports were found by Wedekind et al. (2020), who associated improvements in CP and AA digestibility to the ability of exogenous proteases to hydrolyze Bowman-Birk and Kunitz-trypsin inhibitor proteins. Thus, proteases may improve AA digestibility directly by hydrolysis dietary proteins and indirectly by inhibiting ANFs and consequently facilitating the function of endogenous proteases. Lastly, exogenous protease supplementation can improve digestive functions by improving intestinal morphology (i.e., increasing intestinal villi height) and consequently, absorptive capacity (Park et al., 2020; Song et al., 2022), because can reduce the negative effects of ANFs that are derived from feeding SBM and that can impair digestive and absorptive functions. The protease can also help to release other components like some polysaccharides that can stimulate intestinal health (Poudel et al., 2022).

On the other hand, in phase II, we observed a quadratic increase in digestibility up to 50 g/ton of protease indicating that at higher levels (75 g/ton), protease supplementation could result in negative impact on digestibility. This negative effect may be related to proteolytic imbalance that can result in digestive inflammation (Mariaule et al., 2021). In fact, high inclusion levels of protease have been also associated with damage to the intestinal mucosa (Kumari et al., 2013), which can compromise gut morphology and reduce digestive capacity. However, studies evaluating safety use of exogenous proteases are limited. In addition, enzymes typically interact with one type or a group of similar substrates, resulting in specific enzyme–substrate reaction products (Kiarie et al., 2013). In the case of supplemental proteases, the expected products of dietary protein degradation are absorbable short peptides or AAs. Thus, another possible explanation for the impaired digestibility at 75 g/ton of protease could be the insufficient availability of substrate that could contribute to damage of the intestinal mucosa, particularly considering the type of protease used (serine protease). For serine proteases, the specific substrate is not a complete protein itself, but the peptide bond between AAs.

We consistently observed that the SID of total AA in the SBM diet was higher in younger pigs compared to older pigs with an average of 5.6% increase for indispensable AA. Previous literature in poultry similarly reported that the CP and AA digestibility of rapeseed meal and SBM decreased as the age of broilers increased from 3 to 6 wk (Zuprizal et al., 1992). Barua et al. (2021a) observed the SID coefficients of total AA in sorghum diet were also higher in young broilers compared to old broilers. Engelsmann et al. (2022) verified that SID of 16 AA (Ala, Arg, Asp, Cys, Glu, Gly, His, Ile, Leu, Lys, Met, Phe, Pro, Ser, Thr, and Val) in SBM diets was ~17.91% higher in weaned pigs at 49 d of age compared to 56 d of age and ~12% for overall average of all indispensable AA and ~26.67% for overall average of all dispensable AA.

The difference between younger and older pigs could be related to a decrease in pig efficiency over time. As pigs grow, feed intake increases and feed efficiency decreases (higher feed to gain ratio), which may be related to decreased feed/nutrients retention/utilization rate and decreased nutrient digestibility because of the decreased contact time between the digesta and absorptive surface (Ratanpaul et al., 2021). In addition, it is possible that differences in basal endogenous losses between younger and older pigs could be the reason why we observed statistical differences for SID of AA and not for AID. As part of the digestion process, significant quantities of endogenous protein losses occur, including digestive secretions (bile, gastric, pancreatic, and intestinal secretions), mucoproteins, and desquamated epithelial cells. This inevitable process can influence AA digestibility calculations, therefore proper basal ileal endogenous AA losses estimation is required for obtaining accurate SID coefficients. Commonly, AA digestibly essays occur in younger pigs (<50 kg), but the obtained data are applied to growing pigs of different ages and even sows. Differences in endogenous AA losses as influenced by pig’s age/category may need to be considered to properly calculate SID values. As presented in this study, younger pigs have greater (17.8% increase) endogenous losses of total indispensable AA than older pigs (except for Arg and Lys, Supplementary Table S4). To the best of our knowledge, this study represents the first effort to assess the carryover impact of pig age on the AID and SID of AA for a specific ingredient. In poultry, Barua et al. (2021b) observed differences on basal ileal endogenous flow in broilers of different ages fed wheat and sorghum containing diets. The authors reported that endogenous AA losses were markedly higher on day 7 and, then declined on day 14, and plateaued until day 35 with a further decrease on day 42. Hence, Barua et al. (2021a) related that the SID coefficients were higher in younger broilers (Barua et al., 2021a). These findings support those of the current study.

On the other hand, greater digestibility in younger pigs could be related to total trypsin inhibitor intake. In phase I, pigs were on average 26.5 kg, feed intake was 1.06 kg (4% of BW), and SBM intake 318 g/d (30% SBM inclusion), which resulted in trypsin inhibitor intake per day of 795 mg. In phase II, pigs were on average 57.5 kg, feed intake was 2.3 kg, SBM intake 690 g/d, trypsin inhibitor intake per day was 1,725 mg. Chen et al. (2020) reported that the average value of trypsin inhibitor activity in 192 SBM samples originating from the United States was 3.1 mg/g (equivalent to 5.9 TIU/mg). The SBM used in this study had 4.77 ± 0.67 or TUI = 2.5 mg/g. Therefore, the daily trypsin inhibitor intake was 117% greater in older pigs, which may have contributed to lesser digestibility compared to that in younger pigs. Overall, the exact reasons for the observed effects of age on AA digestibility are likely related to several factors such as digestion, absorption, transport, endogenous losses, feed intake, and trypsin inhibitor intake. Future studies in this line of research are needed to confirm this finding and to better understand the influence of animal age/category on AA digestibility.

In conclusion, dietary protease supplementation at 50 g/ton or 30,000 NFP/kg of feed improved the SID of CP and indispensable AA in SBM by 5.5% and 4.5%, respectively, in both growing and finishing periods. Therefore, protease supplementation can be used as a nutritional strategy to improve protein digestibility, which could contribute to reducing nitrogen emissions from swine production and reducing the feed cost. In addition, while the relative influence of enzyme inclusion on digestibility was consistent across ages, pig age did influence AA digestibility values and may possibly be related to a greater ileal endogenous AA flow in younger pigs. However, this fact warrants further investigation.

## Supplementary Data

Supplementary data are available at *Journal of Animal Science* online.

skae345_suppl_Supplementary_Tables

## Abbreviations:

AA: amino acids
AID: apparent ileal digestibility
BW: body weight
CP: crude protein;
N: nitrogen;
SBM: soybean meal;
SID: standardized ileal digestible
