# Supplementary material for: Effect of protease supplementation on amino acid digestibility of soybean meal fed to growing-finishing pigs in two different ages
Source: J Anim Sci. 2024 Nov 7;102:skae345. doi: 10.1093/jas/skae345 (PMC11630852; doi:10.1093/jas/skae345)
Supplement: skae345_suppl_Supplementary_Tables [file skae345_suppl_supplementary_tables.docx]

**Supplementary table 1**. Experimental diet formulation and calculated composition for the interval period (as-fed basis)^1^.

| **Ingredient, %** | **Inclusion** |
| --- | --- |
| Corn | 73.83 |
| Soybean meal, 46.5% | 23.00 |
| L-Lysine HCl | 0.20 |
| DL-Methionine | 0.00 |
| L- Threonine | 0.06 |
| Monocalcium phosphate | 0.90 |
| Limestone | 1.18 |
| Salt | 0.20 |
| Swine vitamin premix^1^ | 0.05 |
| Swine mineral premix^2^ | 0.15 |
| Swine Larvicide^3^ | 0.13 |
| Titanium oxide | 0.30 |
| **Calculated composition** | |
| ME, Kcal/kg | 3350 |
| Crude Protein, % | 21.0 |
| Crude Fiber, % | 2.50 |
| SID Lys, % | 1.23 |
| SID Arg, % | 0.56 |
| SID Met, % | 0.36 |
| SID, Met + Cys, % | 0.68 |
| SID Thr, % | 0.73 |
| SID Trp, % | 0.20 |
| SID Ile, % | 0.63 |
| SID Leu, % | 1.23 |
| SID Val, % | 0.78 |
| SID His, % | 0.42 |
| SID Phe, % | 0.72 |
| Calcium, % | 0.70 |
| Phosphorus, % | 0.60 |
| ATTD P, % | 0.29 |
| STTD P, % | 0.33 |
| Sodium, % | 0.28 |
| Ca/P | 1.17 |

^1^J & R Distributing Inc. 518 Main Ave, Lake Norden, SD 57248 - USA. Minimum provided per kg of diet: Calcium 55 mg, Vitamin A 11,000 IU, Vitamin D3 1,650 IU, Vitamin E 55 IU; Vitamin B12 0.044 mg, Menadione 4.4 mg, Biotin 0.165 mg, Folic Acid 1.1 mg, Niacin 55 mg, d-Pantothenic Acid 60.5 mg, Vitamin B16 3.3 mg, Riboflavin mg, 9.9 Thiamine 3.3 mg.

^2^J & R Distributing Inc. 518 Main Ave, Lake Norden, SD 57248 - USA. Minimum provided per kg of diet: Copper 16.5 ppm, Mananese 44.1 ppm, Selenium 0.03 ppm, Zinc 165 ppm.

^3^Rabon 7.76 oral larvicide premix active ingredient tetrachlorvinphos manufactured for Elanco US, 2500 Innovation Way, Greenfield, IN46140.

**Supplementary table 2**. Apparent ileal digestibility of amino acids (%) of soybean meal in growing-finishing pigs at different ages^1^.

| **Item** | **Age, days** | | **SEM** | **P-value** |
| --- | --- | --- | --- | --- |
|  | 90 | 140 |  |  |
| CP | 81.71 | 77.55 | 2.640 | 0.119 |
| ***Indispensable AA*** | | | | |
| Arg | 82.13 | 80.19 | 1.578 | 0.223 |
| His | 84.39 | 82.83 | 1.113 | 0.164 |
| Ile | 81.73 | 81.07 | 1.048 | 0.528 |
| Leu | 80.97 | 80.58 | 1.099 | 0.722 |
| Lys | 81.50 | 71.63 | 1.678 | <.0001 |
| Met | 85.78 | 83.57 | 1.202 | 0.070 |
| Met + Cys | 72.39 | 73.64 | 1.747 | 0.475 |
| Phe | 82.55 | 80.86 | 1.046 | 0.111 |
| Thr | 78.84 | 76.73 | 2.013 | 0.298 |
| Trp | 84.07 | 85.03 | 1.017 | 0.336 |
| Val | 75.98 | 74.74 | 1.312 | 0.347 |
| Mean | 80.64 | 78.93 | 1.178 | 0.152 |
| ***Dispensable AA*** | | | | |
| Ala | 58.06 | 53.40 | 2.439 | 0.060 |
| Asp | 79.70 | 78.32 | 1.333 | 0.305 |
| Cys | 58.99 | 63.57 | 2.307 | 0.051 |
| Glu | 87.90 | 84.96 | 1.231 | 0.020 |
| Ser | 73.08 | 77.64 | 1.425 | 0.002 |
| Tyr | 82.01 | 78.80 | 1.399 | 0.025 |
| Mean | 67.30 | 66.42 | 2.003 | 0.661 |
| Total AA mean | 62.43 | 60.01 | 2.272 | 0.290 |

^1^In phase I, the pig BW ranged from 23 to 30 kg, with an average age of 90 days. In phase II, the pig BW ranged from 50 to 65 kg, with an average age of 140 days.

**Supplementary table 3**. Standardized ileal digestibility of amino acids (%) of soybean meal in growing-finishing pigs at different ages^1^.

| **Item** | **Age, days** | | **SEM** | **P-value** |
| --- | --- | --- | --- | --- |
|  | 90 | 140 |  |  |
| CP | 84.56 | 79.81 | 1.831 | 0.011 |
| ***Indispensable AA*** | | | | |
| Arg | 88.81 | 86.83 | 1.336 | 0.142 |
| His | 88.19 | 84.24 | 0.996 | <.0001 |
| Ile | 86.67 | 81.32 | 1.036 | <.0001 |
| Leu | 84.46 | 80.74 | 1.036 | 0.001 |
| Lys | 85.85 | 77.32 | 1.754 | <.0001 |
| Met | 89.87 | 83.76 | 1.086 | <.0001 |
| Met + Cys | 78.15 | 75.34 | 1.638 | 0.091 |
| Phe | 86.29 | 81.01 | 0.959 | <.0001 |
| Thr | 81.01 | 76.98 | 1.945 | 0.041 |
| Trp | 90.84 | 85.03 | 0.946 | <.0001 |
| Val | 81.57 | 76.33 | 1.203 | <.0001 |
| Mean | 85.79 | 81.22 | 1.151 | <.0001 |
| ***Dispensable AA*** | | | | |
| Ala | 66.10 | 59.07 | 2.652 | 0.010 |
| Asp | 82.97 | 78.55 | 1.244 | 0.001 |
| Cys | 66.43 | 67.19 | 2.160 | 0.727 |
| Glu | 90.23 | 83.65 | 1.270 | <.0001 |
| Ser | 80.36 | 81.51 | 1.394 | 0.410 |
| Tyr | 87.06 | 81.61 | 1.322 | <.0001 |
| Mean | 71.02 | 70.89 | 2.104 | 0.949 |
| Total AA mean | 71.60 | 67.46 | 2.364 | 0.084 |

^1^In phase I, the pig BW ranged from 23 to 30 kg, with an average age of 90 days. In phase II, the pig BW ranged from 50 to 65 kg, with an average age of 140 days.

**Supplementary table 4.** Basal ileal endogenous amino acid flows (g per 100 g DM) of growing-finishing pigs at different ages^1^.

| **Item** | **Age, days** | | **SEM** | **P-value** |
| --- | --- | --- | --- | --- |
|  | 90 | 140 |  |  |
| ***Indispensable AA*** | | | | |
| Arg | 0.67 | 0.68 | 0.111 | 0.887 |
| His | 0.20 | 0.15 | 0.027 | 0.108 |
| Ile | 0.35 | 0.27 | 0.074 | 0.305 |
| Leu | 0.60 | 0.43 | 0.122 | 0.197 |
| Lys | 0.54 | 0.69 | 0.112 | 0.181 |
| Met | 0.10 | 0.07 | 0.019 | 0.152 |
| Phe | 0.39 | 0.27 | 0.076 | 0.136 |
| Thr | 0.59 | 0.43 | 0.064 | 0.023 |
| Trp | 0.13 | 0.09 | 0.019 | 0.031 |
| Val | 0.49 | 0.34 | 0.076 | 0.062 |
| Total indispensable AA | 4.04 | 3.43 | 0.641 | 0.349 |
| ***Dispensable AA*** | | | | |
| Ala | 0.67 | 0.59 | 0.101 | 0.415 |
| Asp | 0.88 | 0.62 | 0.178 | 0.162 |
| Cys | 0.20 | 0.15 | 0.033 | 0.194 |
| Glu | 1.10 | 0.76 | 0.237 | 0.171 |
| Ser | 0.54 | 0.40 | 0.060 | 0.028 |
| Tyr | 0.27 | 0.21 | 0.042 | 0.187 |
| Total dispensable AA | 11.28 | 11.27 | 1.298 | 0.998 |
| Total AA | 15.32 | 14.70 | 1.796 | 0.734 |

^1^In phase I, the pig BW ranged from 23 to 30 kg, with an average age of 90 days. In phase II, the pig BW ranged from 50 to 65 kg, with an average age of 140 days.
